# The *Drosophila* MCPH1-B isoform is a substrate of the APC^Cdh1^ E3 ubiquitin ligase complex

**DOI:** 10.1242/bio.20148318

**Published:** 2014-06-27

**Authors:** Sarah G. Hainline, Jamie L. Rickmyre, Leif R. Neitzel, Laura A. Lee, Ethan Lee

**Affiliations:** Department of Cell and Developmental Biology, Vanderbilt University Medical Center, Nashville, TN 37232-8240, USA; * Present address: Sarah Cannon Research Institute, Nashville, TN 37203, USA.

**Keywords:** Anaphase-Promoting Complex, *Drosophila*, MCPH1, Ubiquitination, *Xenopus* egg extract

## Abstract

The Anaphase-Promoting Complex (APC) is a multi-subunit E3 ubiquitin ligase that coordinates progression through the cell cycle by temporally and spatially promoting the degradation of key proteins. Many of these targeted proteins have been shown to play important roles in regulating orderly progression through the cell cycle. Using a previously described *Drosophila* in vitro expression cloning approach, we screened for new substrates of the APC in *Xenopus* egg extract and identified *Drosophila* MCPH1 (dMCPH1), a protein encoded by the homolog of a causative gene for autosomal recessive primary microcephaly in humans. The dMCPH1-B splice form, but not the dMCPH1-C splice form, undergoes robust degradation in *Xenopus* interphase egg extract in a Cdh1-dependent manner. Degradation of dMCPH1-B is controlled by an N-terminal destruction box (D-box) motif as its deletion or mutation blocks dMCPH1-B degradation. dMCPH1 levels are increased in *Drosophila morula* (*APC2*) mutant embryos, consistent with dMCPH1 being an APC substrate in vivo. Using a purified, reconstituted system, we show that dMCPH1-B is ubiquitinated by APC^Cdh1^, indicating that the effect of APC on dMCPH1-B ubiquitination and degradation is direct. Full-length human MCPH1 (hMCPH1) has been predicted to be an APC substrate based on its interaction with the APC subunit Cdc27. We were not able to detect changes in hMCPH1 levels during the cell cycle in cultured human cells. Overexpression of hMCPH1 (or dMCPH1-B) in developing *Xenopus* embryos, however, disrupts cell division, suggesting that proper regulation of hMCPH1 and dMCPH1-B activity plays a critical role in proper cell-cycle progression.

## INTRODUCTION

The Anaphase-Promoting Complex (APC) is a multi-subunit E3 ubiquitin ligase that catalyzes ubiquitin-mediated proteasomal degradation of target proteins. A major function of the APC is to promote degradation of key cell-cycle proteins so as to coordinate orderly progression through the cell cycle (Peters, 2006). Human and yeast APC are each composed of 14–15 identified subunits and two primary co-activators, Cdc20 and Cdh1 (Kulkarni et al., 2013). Destruction of APC substrates is required in eukaryotes for the initiation of anaphase and exit from mitosis. Cdc20 associates with the APC in early mitosis, leading to the destruction of proteins that control the onset of anaphase, whereas Cdh1 promotes degradation of APC substrates that control late mitosis and the following G1 phase. These co-activators provide APC substrate specificity by facilitating the recognition of specific destruction motifs (e.g. degrons) such as the D-box (RxxLxxxxN) or KEN box (Lys–Glu–Asn) (King et al., 1996; Min and Lindon, 2012; Pfleger and Kirschner, 2000). Mutations of these motifs block the recognition of the protein by the APC, preventing their APC-mediated destruction.

*Xenopus* egg extract contains many of the components necessary for ubiquitin-mediated degradation such as E1, E2, and E3 enzymes, ubiquitin, and the proteasome. Moreover, biochemical regulation of APC^Cdc20^- and APC^Cdh1^-mediated degradation has been well studied and characterized in this system. *Xenopus* egg extract lacks Cdh1, and Cdc20 is the primary activator of APC (Lorca et al., 1998). Addition of exogenous human Cyclin B lacking its N-terminal D-box (CycBΔ90) to interphase *Xenopus* egg extract drives the extract into mitosis and promotes the degradation of APC^Cdc20^ substrates (Glotzer et al., 1991). Addition of exogenous Cdh1 to interphase *Xenopus* egg extract similarly promotes the degradation of APC^Cdh1^ substrates (Pfleger and Kirschner, 2000).

The in vitro expression cloning (IVEC) strategy involves generating [^35^S]methionine-labeled proteins by in vitro-coupled transcription and translation of small, random pools of cDNAs; these radiolabeled proteins can then be used for biochemical screening in a powerful approach that allows for rapid isolation of relevant cDNAs corresponding to “hits” in the screen (King et al., 1997). IVEC has been successfully used in *Xenopus* egg extract to identify important APC substrates such as Geminin, Securin, Xkid, Tome-1, and Sororin (Ayad et al., 2003; Funabiki and Murray, 2000; McGarry and Kirschner, 1998; Rankin et al., 2005; Zou et al., 1999). A weakness of the original IVEC strategy, however, is that, depending on the cDNA library being used, certain genes are over-represented whereas other genes are under-represented in the library. Thus, the same substrate is often identified over and over again, and substantial screening is necessary to identify relevant rare clones. Furthermore, the pools of cDNAs used for IVEC screening must be deconvoluted in order to isolate single hits as the identities of the clones in the pools are unknown.

To overcome these limitations, we previously modified the IVEC methodology to generate radiolabeled protein pools from Release 1 of the *Drosophila* Gene Collection (DGC), an annotated unigene set of 5,849 full-length cDNA clones representing 43% of the fly genome (Lee et al., 2005; Stapleton et al., 2002). Clones were individually arrayed in 17 × 384-well plates, and in vitro transcription and translation was performed on small pools containing equivalent amounts of cDNA (or mRNA) for each gene. This *Drosophila* IVEC (DIVEC) approach has allowed for efficient genome-scale screening to identify substrates of the Pan Gu kinase and binding partners of p53 (Lee et al., 2005; Lunardi et al., 2010).

Given the conservation across phyla between cell cycle proteins, we herein applied the DIVEC approach to perform a biochemical screen for APC substrates in *Xenopus* interphase egg extract and identified *Drosophila* Microcephalin (dMCPH1) as a candidate. Human MCPH1 (hMCPH1) is a causative gene of autosomal recessive primary microcephaly (MCPH), a neurodevelopment disorder characterized by reduced brain size (Jackson et al., 2002; Woods et al., 2005). In humans, MCPH1 has been shown to prevent premature mitotic entry by regulating centrosomal recruitment of Chk1 at the G2/M transition as well as premature chromosome condensation by negatively regulating the activity of condensin II (Gruber et al., 2011; Tibelius et al., 2009; Trimborn et al., 2006; Yamashita et al., 2011). hMCPH1 has also been reported to have several functions in the DNA damage response (Gavvovidis et al., 2012; Lin et al., 2005; Peng et al., 2009; Rai et al., 2006; Tibelius et al., 2009; Trimborn et al., 2006; Yamashita et al., 2011; Yang et al., 2008). We previously reported that *Drosophila* syncytial embryos derived from *mcph1*-null females exhibit Chk2-mediated mitotic arrest in response to damaged or incompletely replicated DNA (Rickmyre et al., 2007). Because *mcph1* mutants contain an intact DNA checkpoint, and MCPH1 has been shown to regulate premature chromosome condensation in other systems, we previously proposed that dMCPH1 prevents accumulation of DNA damage by delaying chromosome condensation until DNA replication is completed. Although MCPH1 is reported to function in multiple cellular processes, its regulation is not well understood. In this report, we demonstrate that dMCPH1 is a substrate of the critical cell cycle regulator, APC^Cdh1^.

## MATERIALS AND METHODS

### cDNA clones and mutagenesis

cDNA clones encoding dMCPH1-B (clone LD42241), dMCPH1-C (clone LP15451), or p78 (GH13229) were obtained from the *Drosophila* Gene Collection Release 1 or the *Drosophila* Genomics Resource Center (Indiana University, Bloomington, IN), respectively. cDNA clones encoding hMCPH1, Cyclin B, NT-Cyclin B, Mos, Luciferase, and GFP were gifts from Marc Kirschner's lab (Harvard Medical School, Boston, MA). dMCPH1-B and dMCPH1-C were subcloned into vector pCS2 for in vitro transcription and translation reactions. dMCPH1-B^ΔN^, dMCPH1-B^DboxMut^, and dMCPH1-B^1–64^ were generated from CS2-dMCPH1-B by mutagenesis to remove the first 40 amino acids, replace amino acids 36–40 with alanines, or remove the last 762 amino acids, respectively. dMCPH1-B, dMCPH1-B^DboxMut^, and hMCPH1 were also subcloned into pCS2 derivatives encoding six N- or C-terminal Myc tags.

### DIVEC screen and APC degradation assay

*Xenopus* interphase egg extract was prepared as previously described (Pfleger and Kirschner, 2000). Baculoviruses encoding human His6-tagged CDH1 and His6-tagged Cyclin BΔ90 (gifts from Marc Kirschner's lab) were expressed in *Sf9* cells by baculovirus infection and purified over nickel beads. For the DIVEC screen, radiolabeled protein pools were generated from pools of cDNAs from the *Drosophila* Gene Collection Release 1 by transcription and translation in reticulocyte lysates using a Gold TNT T7 kit according to the manufacturer's protocol (Promega, Madison, WI) as previously described (Lee et al., 2005). The identity of positive clones was confirmed by DNA sequencing.

For testing individual proteins in the APC degradation assay, 1 µl of radiolabeled protein was added to 10 µl of *Xenopus* egg extract supplemented with energy mix (1 mM HEPES, pH 7.7, 1 mM ATP, 10 mM creatine phosphate, and 1 mM MgCl_2_) and 10 µg/ml ubiquitin. Egg extract was incubated with *Xenopus* Buffer control (100 mM KCl, 1 mM MgCl_2_, 0.1 mM CaCl_2_, 10 mM HEPES, 50 mM sucrose, 5 mM EGTA), His6-Cyclin BΔ90 (60 µg/ml), or His6-CDH1 (0.4 nM) prior to starting the reaction with addition of radiolabeled proteins, and reactions were allowed to proceed at room temperature as previously described (Ayad et al., 2003). All radiolabeled, in vitro-translated protein migrated at the expected size as assessed by SDS-PAGE/autoradiography. For radiolabeled degradation assays, loading controls were not necessary as equivalent volumes (0.5 µl) were removed at the indicated times for processing by SDS-PAGE/autoradiography. NT-Cyclin B peptide 100 µM was prepared as previously described (Pfleger and Kirschner, 2000). Pixel intensity measurements of autoradiograms were performed using ImageJ and statistical analysis was performed using the paired equal variance two-tailed t-test.

### *Drosophila* stocks, embryo lysates, and immunoblotting

Flies were maintained at 25°C using standard techniques (Greenspan, 2004). *morula* stocks (*mr^1^* and *mr^2^*) were gifts from T. Orr-Weaver (Whitehead Institute, Cambridge, MA) (Reed and Orr-Weaver, 1997). *y^1^ w^1118^* flies were used as the “wild-type” stock. Embryo lysates were made by homogenizing embryos (0–1 hour) in urea sample buffer (100 mM Tris, pH 7.6, 8 M urea, 2% SDS, 5% β-mercaptoethanol, and 5% Ficoll). Lysates were analyzed by SDS-PAGE and immunoblotting using standard techniques. Primary antibodies used included guinea pig anti-MCPH1 (1:200) (Rickmyre et al., 2007); mouse anti-Cyclin B (1:200, F2F4, Developmental Studies Hybridoma Bank, Iowa City, IA); and mouse anti-α-tubulin (1:5000, DM1α, Sigma–Aldrich, St Louis, MO). HRP-conjugated secondary antibodies were used to detect primary antibodies by chemiluminescence.

### In vitro ubiquitination assay

APC was purified by immunoprecipitation of Cdc27 from *Xenopus* interphase egg extract using Protein G Sepharose beads (GE Healthcare Life Sciences, Pittsburgh, PA) and anti-Cdc27 antibodies (AF3.1; Santa Cruz Biotechnology, Dallas, TX) as previously described (Wei et al., 2004). For each ubiquitination reaction, 5 µl of APC-bound beads was incubated with 0.75 µM purified E1 (Boston Biochem, Cambridge, MA), 2 µM His-UbcH10 (Boston Biochem), 7.5 mg/ml ubiquitin (Boston Biochem), 0.5 µl 20× Energy Regeneration Mix (2 mg/ml creatine phosphokinase, 20 mM ATP, 200 mM Creatine Phosphate, 20 mM HEPES, 20 mM MgCl_2_, 0.1% BSA), 5 µM ubiquitin aldehyde (Boston Biochem), and 10 mM DTT. 1 µl of in vitro transcription/translation reaction product and 0.4 nM His-Cdh1 or equal volume of Cdh1 dialysis buffer was incubated in each reaction for 90 minutes. Reaction products were separated by SDS-PAGE and visualized by autoradiography.

### *Xenopus* embryo injection, immunostaining, and immunoblotting

Capped mRNA encoding Mos, GFP, hMCPH1, dMCPH1-B, or dMCPH1-B^DboxMut^ was generated by in vitro transcription reactions using the mMessage mMachine kit per manufacturer's instructions (Life Technologies, Carlsbad, CA). Embryos were injected at the 2- or 4-cell stage with 2 ng of RNA and fixed in MEMFA (100 µM MOPS pH 7.4, 2 mM EGTA, 1 mM MgSO_4_, and 3.7% formaldehyde) after 4 hours. After fixation, embryos were washed 2× in PBS and dehydrated stepwise (1 hour/step) in 75% PBS/25% methanol, 50% PBS/50% methanol, and 100% methanol and stored at 4°C. The percentage of injected embryos exhibiting cell-cycle defects was quantified and statistical analysis was performed using the Fisher exact test.

For tubulin staining, MEMFA-fixed embryos (in 100% methanol) were bleached in 10% H_2_O_2_/67% methanol for 8 hours at room temperature. Bleached embryos were rehydrated (1 hour/step) in 50% methanol/50% TBS (155 mM NaCl, 10 mM Tris-HCl pH 7.5), 25% methanol/75% TBS, and finally 100% TBST (TBS plus 0.1% Triton X-100). Embryos were then blocked in WMBS (TBS plus 10% fetal bovine serum and 5% DMSO) for 1 hour. Mouse anti-α-tubulin (DM1α, 1:500, Sigma), RNAse A (1 mg/ml), and propidium iodide (2 µg/ml) were then added and embryos were incubated overnight at 4°C. Embryos were washed 5× (1 hour each) with TBST and incubated in WMBS with RNAse A, propidium iodide, and Cy2-conjugated secondary antibodies (1:500, Sigma). Embryos were washed 5× (1 hour each) with TBST, placed in MatTek dishes (Ashland, MA), and imaged using a Leica TCS SP5 inverted confocal microscope (Buffalo Grove, IL).

For immunoblotting, capped mRNA encoding C-terminally Myc-tagged hMCPH1, dMCPH1-B, or dMCPH1-B^DboxMut^ was generated, and 1 ng of RNA was injected into each cell of a two-cell staged *Xenopus* embryo. At 4 hours post-injection, the embryos were lysed in 6× Sample Buffer (300 mM Tris pH 6.8, 12% w/v SDS, 30% w/v glycerol, 600 mM DTT, and 0.01% w/v bromophenol blue). One quarter of each lysate was analyzed by SDS-PAGE and immunoblotting using standard techniques. Primary antibodies used included mouse anti-Myc-tag (1:500, 9E10) and mouse anti-α-tubulin (1:2000, DM1α, Sigma–Aldrich, St Louis, MO). HRP-conjugated secondary antibodies were used to detect primary antibodies by chemiluminescence.

All *Xenopus* experiments conform to institutional and national animal welfare policies.

### Cell synchronization

24 hours after plating HeLa cells on 150 mm dishes at 20% confluency, cells were treated with nocodazole (25 ng/ml) for 13 hours. Plates were firmly tapped to loosen the rounded, mitotic cells from the dish. Cells were then collected by centrifugation for 5 minutes, and washed 3 times in fresh serum-free medium. After the final wash, cells were resuspended in medium containing 10% FBS and plated at 50% confluency in 6-well dishes. Cells were collected every 2 hours by removing medium, washing in PBS, treating with 100 µl 0.25% trypsin-EDTA, and collecting in 1 ml medium. Collected cells were washed once in PBS and lysed in non-denaturing lysis buffer (50 mM Tris-HCl, pH 7.4, 300 mM NaCl, 5 mM EDTA, 1% Triton X-100). Lysates were analyzed by SDS-PAGE and immunoblotting using standard techniques. Antibodies used were rabbit anti-hMCPH1 (D38G5, 1:100, Cell Signaling Technology, Danvers, MA), rabbit anti-Cdk1 (1:4000, Millipore, Billerica, MA), rabbit anti-Cyclin A (H-432, 1:500, Santa Cruz), rabbit anti-Cyclin B1 (H-20, 1:500, Santa Cruz), and rabbit anti-p27 (C-19, 1:100, Santa Cruz). HRP-conjugated secondary antibodies and chemiluminescence were used to detect primary antibodies.

## RESULTS

### DIVEC screen for APC substrates

**Fig. 1.**
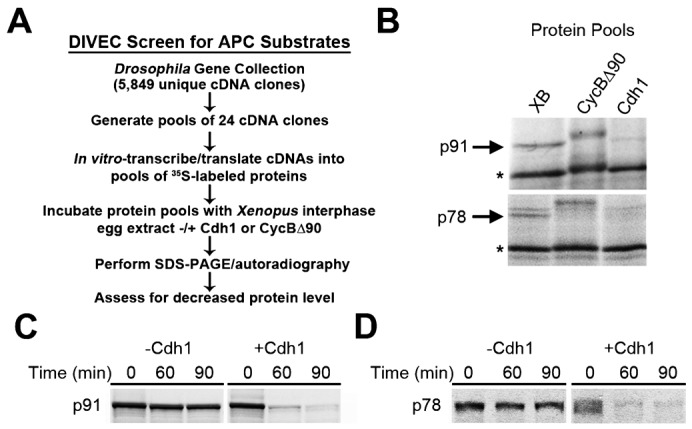
A *Drosophila* In Vitro Expression Cloning (DIVEC) screen identifies two novel APC substrates. (A) Schematic of the DIVEC screen strategy to identify APC substrates. ^35^S-radiolabeled proteins were produced from pools of cDNA clones from the *Drosophila* Gene Collection Release 1 as previously described (Lee et al., 2005). Radiolabeled protein pools were incubated in *Xenopus* interphase egg extract supplemented with *Xenopus* buffer (XB), non-degradable Cyclin B (CycBΔ90), or Cdh1. Reaction products were analyzed by SDS-PAGE and autoradiography to identify proteins degraded via APC-Cdc20 or APC-Cdh1. (B) p91 and p78 are candidate APC substrates. Autoradiogram of two protein pools containing p91 and p78. Both p91 and p78 exhibited an upward electrophoretic mobility shift in CycBΔ90-supplemented (mitotic) extract and decreased band intensity in Cdh1-supplemented extract. Asterisks mark proteins in the pools that did not exhibit decreased intensity in the supplemented extract and therefore served as negative controls. (C,D) Retesting of radiolabeled p91 and p78 (prepared from individual cDNA clones) by incubation in *Xenopus* interphase egg extract in the presence of Cdh1 confirmed that the clones encode putative APC substrates.

In order to identify APC^Cdc20^ or APC^Cdh1^ substrates using DIVEC, bacterial stocks containing cDNA clones from the *Drosophila* Gene Collection Release 1 were individually grown and their plasmids purified and pooled (Fig. 1A). Pooled clones (24 clones/pool) were used to generate radiolabeled proteins in rabbit reticulocyte lysate as previously described (Lee et al., 2005). To test proteins for their capacity to undergo APC^CDC20^- or APC^Cdh1^-mediated degradation, protein pools were incubated in *Xenopus* interphase egg extract supplemented with *Xenopus* buffer (XB), human CycBΔ90, or Cdh1. Candidate APC substrates were identified by their decreased band intensity after incubation in CycBΔ90 or Cdh1-supplemented extract relative to the buffer control as revealed by SDS-PAGE and autoradiography.

We identified two candidate substrates of APC in *Xenopus* egg extract using the DIVEC approach (Fig. 1B). We initially named these candidates “p78” and “p91” based on their apparent SDS-PAGE mobility. In the primary screen that involved the use of radiolabeled protein pools, both candidates were stable in the presence of XB and CycBΔ90 (mitotic extract containing activated APC^Cdc20^), but they degraded in *Xenopus* egg extract supplemented with Cdh1, suggesting that they are substrates of APC^Cdh1^ and not APC^Cdc20^. In addition, both candidates exhibited decreased mobility on SDS-PAGE when incubated in Cyclin BΔ90-supplemented (mitotic) extract, suggesting that they may be phosphorylated during mitosis.

The corresponding cDNA clones for the two candidate substrates were identified based on the predicted molecular weights of their encoded proteins and retesting in the degradation assay. We confirmed that the protein products generated by in vitro transcription and translation of these individual cDNA clones were degraded in Cdh1-supplemented *Xenopus* egg extract (Fig. 1C,D). p91 is encoded by clone LD43341 and corresponds to the *Drosophila mcph1* gene (Brunk et al., 2007; Rickmyre et al., 2007). p78 is encoded by clone GH13229 and corresponds to *CG32982*, an uncharacterized *Drosophila* gene. Cyclin B, a well-characterized APC substrate, was not identified in our screen because it is not present in the *Drosophila* Gene Collection Release 1. Radiolabeled Cyclin B, however, was used as a positive control in our screen and was shown to degrade in both mitotic (activated APC^Cdc20^) and Cdh1-supplemented interphase *Xenopus* egg extract (data not shown).

### *Drosophila* MCPH1-B stability is regulated by APC

We previously identified a requirement for dMCPH1 during early embryogenesis in *Drosophila* (Rickmyre et al., 2007). Two distinct isoforms of *Drosophila* MCPH1 (referred to as MCPH1-B and MCPH1-C) are produced by alternative splicing (Rickmyre et al., 2007). Both isoforms are present in larval brains and imaginal discs. *Drosophila* MCPH1-B (dMCPH1-B) is predominantly expressed in the ovaries and syncytial embryos, whereas MCPH1-C (dMCPH1-C) is expressed primarily in the testes. The two isoforms differ primarily at their N- and C-termini. dMCPH1-B contains an additional 47 amino acids at its N-terminal end and lacks 200 amino acids at its C-terminal end when compared to the dMCPH1-C isoform (supplementary material Fig. S1A). Both isoforms contain an N-terminal BRCT domain. Only dMCPH1-C, however, contains an additional pair of BRCT domains at its C-terminal end.

**Fig. 2.**
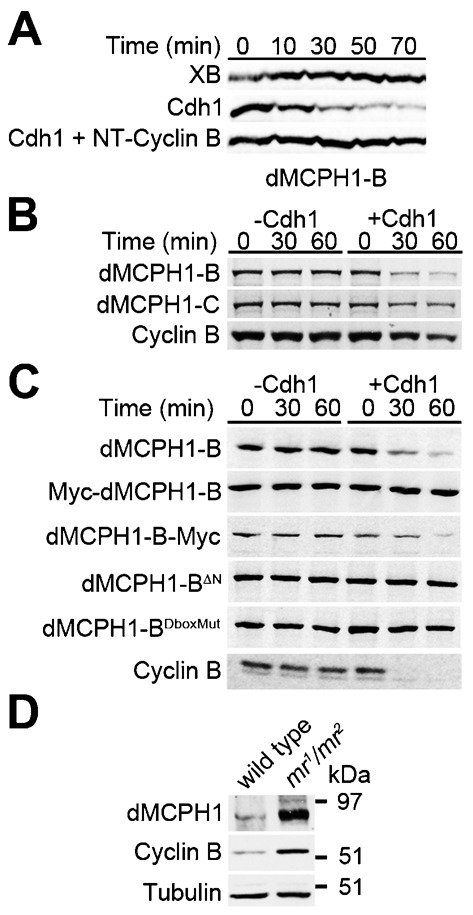
dMCPH1-B stability is regulated by APC. (A) dMCPH1 degradation in *Xenopus* egg extract is stimulated by Cdh1. Radiolabeled dMCPH1-B was incubated in *Xenopus* interphase egg extract supplemented with XB (buffer control), Cdh1, or Cdh1 plus an N-terminal Cyclin B peptide (NT-Cyclin B). (B) Degradation of dMCPH1-C in *Xenopus* egg extract. Radiolabeled dMCPH1-B, dMCPH1-C, or Cyclin B was incubated in *Xenopus* interphase egg extract in the absence or presence of Cdh1. See supplementary material Fig. S1B for quantification of gel band intensities. (C) A free N-terminal end of dMCPH1-B, which contains a putative D-box, is required for its Cdh1-stimulated degradation in *Xenopus* egg extract. Wild-type dMCPH1-B and a C-terminally Myc-tagged version (dMCPH1-B-Myc) degraded in *Xenopus* egg extract in the presence of Cdh1. In contrast, an N-terminally Myc-tagged version (Myc-dMCPH1-B), an N-terminal deletion mutant (dMCPH1-B^ΔN^), or an N-terminal D-box mutant (dMCPH1-B^DboxMut^) failed to degrade in Cdh1-stimulated *Xenopus* egg extract. (D) Immunoblot analysis of dMCPH1, Cyclin B, and alpha-Tubulin levels in lysates derived from embryos (0–1 hour) of *APC2* mutant (*mr^1^*/*mr^2^*) females indicate that dMCPH1 levels are increased in the mutant embryos compared to wild-type embryos.

We identified the B isoform of dMCPH1 as a hit in our DIVEC screen for APC substrates. To demonstrate that the degradation of dMCPH1-B in *Xenopus* egg extract was specific to APC^Cdh1^ activity, we tested whether Cdh1-mediated degradation of dMCPH1-B in *Xenopus* interphase egg extract could be inhibited by addition of an N-terminal peptide of Cyclin B (NT-Cyclin B) containing a functional D-box (Fig. 2A). NT-Cyclin B is degraded in Cdh1-supplemented egg extract and competitively blocks APC^Cdh1^-mediated degradation of Cdc20 (Pfleger and Kirschner, 2000). Similarly, if dMCPH1-B degradation in Cdh1-supplemented *Xenopus* interphase egg extract were mediated by APC^Cdh1^, addition of excess NT-Cyclin B should inhibit its degradation. Consistent with this model, we found that addition of NT-Cyclin B potently blocked dMCPH1-B degradation in Cdh1-supplemented extract (Fig. 2A).

We next asked if the MCPH1-C isoform is also a substrate of APC^Cdh1^. We incubated radiolabeled dMCPH1-C in *Xenopus* interphase egg extract in the absence or presence of Cdh1 and assessed its levels after 30 and 60 minutes by performing SDS-PAGE/autoradiography (Fig. 2B; supplementary material Fig. S1B). For dMCPH1-B and Cyclin B (positive control), we detected robust turnover in Cdh1-supplemented *Xenopus* interphase egg extract. Although we detected statistically significant Cdh1-mediated degradation for dMCPH1-C, it was not nearly as robust as that of dMCPH1-B or Cyclin B.

During our characterization of dMCPH1-B degradation, we found that an N-terminally Myc-tagged, but not a C-terminally Myc-tagged, version of dMCPH1-B degraded in *Xenopus* interphase egg extract (Fig. 2C), suggesting that the N-terminal Myc-tag might mask a nearby degron. These findings were consistent with a model in which the first 47 amino acids of dMCPH1-B that is not shared with dMCPH1-C contains the relevant degron that mediates degradation by APC^Cdh1^. To test this possibility, we generated an N-terminal truncation mutant of dMCPH1-B (dMCPH1-B^ΔN^) in which the first 40 amino acids was deleted. We found that this mutant was stable in Cdh1-supplemented extract, indicating that the N-terminal end of dMCPH1-B contains a degron necessary for APC^Cdh1^-mediated degradation (Fig. 2C).

We identified a putative D-box motif (RRPLHDSN) within the first 40 amino acids of dMCPH1-B and generated a mutant in which the first four amino acids of this sequence were replaced with alanines (dMCPH1-B^DboxMut^). We found that, in contrast to the wild-type protein, dMCPH1-B^DboxMut^ was stable in Cdh1-supplemented extract (Fig. 2C). These data indicate the D-box sequence found within the N-terminal 40 amino acids of dMCPH1-B mediates its APC^Cdh1^-dependent degradation.

Mutants of the *Drosophila morula* (*mr*) gene, which encodes the homolog of the vertebrate APC2 subunit of APC, have increased levels of Cyclin B due to reduced APC activity (Reed and Orr-Weaver, 1997). Syncytial embryos laid by females transhetersozygous for *mr^1^* and *mr^2^* alleles (*mr^1^/mr^2^*) arrest in mitosis shortly after a few cell cycles. dMCPH1-B is primarily expressed in syncytial embryos (Brunk et al., 2007; Rickmyre et al., 2007). If dMCPH1-B were an APC substrate, we reasoned that its levels should be increased in *morula* mutant flies. To test this possibility, we prepared lysates from 0–1 hour syncytial embryos derived from wild-type or *mr^1^/mr^2^* females and assessed endogenous dMCPH1, Cyclin B (positive control), and alpha-tubulin (loading control) levels by immunoblotting (Fig. 2D). Embryos derived from *mr^1^/mr^2^* females had increased levels of both dMCPH1-B and Cyclin B compared to wild type, suggesting that dMCPH1-B is an APC substrate in vivo. *mr^1^/mr^2^*-derived embryos also contain dMCPH1, which exhibits slower mobility on SDS-PAGE (Fig. 2D). Because *mr^1^/mr^2^*-derived embryos are reported to arrest in mitosis, it is possible that this form of dMCPH1 is the result of mitotic phosphorylation.

### dMCPH1-B is ubiquitinated by APC

**Fig. 3.**
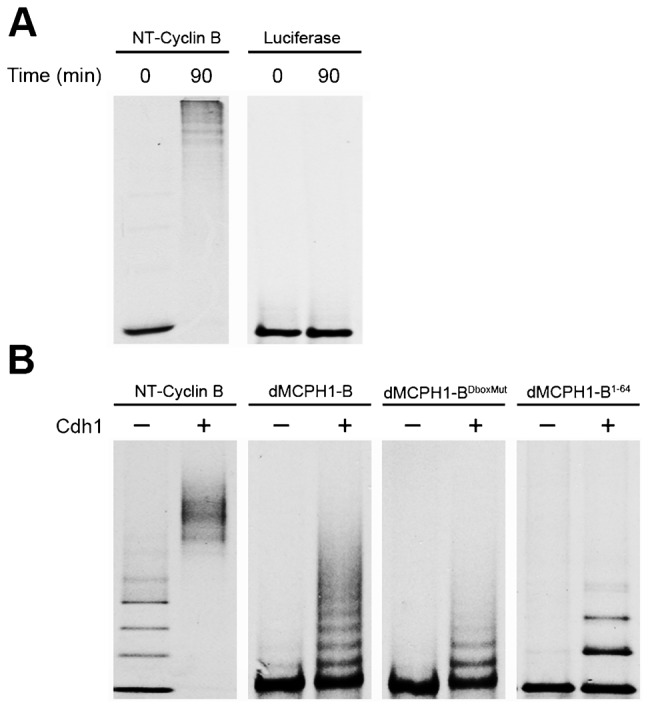
dMCPH1-B is ubiquitinated by APC in vitro. (A) Establishment of an in vitro APC ubiquitination assay. Radiolabeled N-terminal peptide of Cyclin B (NT-Cyclin B) and Luciferase protein were incubated in a reaction containing APC purified from *Xenopus* interphase egg extract, purified human E1, His-UbcH10, His-Cdh1, ubiquitin, and an energy regeneration system. Reactions were terminated by addition of sample buffer followed by SDS-PAGE/autoradiography. (B) dMCPH1-B is an in vitro substrate of APC^Cdh1^, and its ubiquitination is mediated in large part by its N-terminal D-box. Radiolabeled NT-Cyclin B, dMCPH1-B, dMCPH1-B^Dboxmut^, and the N-terminal 64 amino acid fragment of dMCPH1-B (dMCPH1-B^1–64^) were incubated in the APC ubiquitination assay and reaction products assessed by SDS-PAGE/autoradiography.

We next sought to determine whether dMCPH1-B is a direct substrate of APC^Cdh1^ using a purified system as previously described (King et al., 1995; Pfleger and Kirschner, 2000). The APC was purified from *Xenopus* interphase egg extract by immunoprecipitation using an antibody against the Cdc27 subunit. Purified APC was then used for in vitro ubiquitination reactions containing recombinant human E1, E2 (UbcH10), Cdh1, and ubiquitin. The radiolabeled NT-Cyclin B peptide (positive control) was polyubiquinated as evidenced by the presence of higher molecular weight laddering on SDS-PAGE (Fig. 3A). In contrast, no laddering was detected for firefly luciferase (negative control).

We next tested whether dMCPH1-B was ubiquitinated in our purified system and whether addition of Cdh1 would enhance ubiquitination. We found that ubiquitination of dMCPH1-B and NT-Cyclin B (positive control) was dramatically enhanced in the presence of Cdh1 in our reconstituted ubiquitination system, consistent with dMCPH1-B being an APC^Cdh1^ substrate (Fig. 3B). Ubiquitination of the D-box mutant, dMCPH1-B^DboxMut^, was observed in the presence of Cdh1, albeit at a much reduced level (Fig. 3B). This phenomenon has been observed with other APC substrates in the purified system (Araki et al., 2005; Fang et al., 1998b; Pfleger and Kirschner, 2000), and the low level of ubiquitination observed likely reflects the fact that the purified system lacks many regulatory proteins present in an extract or cell. To further confirm that the N-terminal end of dMCPH1-B contains a functional D-box, we showed that the first 64 amino acids of dMCPH1-B (dMCPH1-B^1–64^) was ubiquitinated in the purified system and that ubiquitination was enhanced in the presence of Cdh1 (Fig. 3B). These results indicate that dMCPH1-B is a direct substrate of APC^Cdh1^ in vitro and that the N-terminal D-box of dMCPH1-B plays a major role in mediating its ubiquitination by APC.

### Steady state-levels of hMCPH1 do not change in a cell cycle-dependent manner in cultured human cells

Human MCPH1 (hMCPH1) contains one N-terminal and two C-terminal BRCT domains and is more similar to dMCPH1-C in organization than dMCPH1-B (supplementary material Fig. S1A). Although hMCPH1 lacks an N-terminal degron similar to dMCPH1-B, it contains several putative D-boxes and a candidate KEN box. To determine if hMCPH1 is also degraded via APC^Cdh1^, radiolabeled hMCPH1 was incubated in *Xenopus* interphase egg extract in the absence or presence of Cdh1. In contrast to dMCPH1-B, hMCPH1 did not degrade in Cdh1-supplemented extract (supplementary material Fig. S2A). The observed doublet is consistent with an alternative translation initiation downstream (35 amino acids) of the canonical start site using the rabbit reticulocyte translation system. It is possible that the incapacity of *Xenopus* interphase egg extract to support hMCPH1 degradation by APC^Cdh1^ is due to differences between the amphibian and human systems.

We next assessed the steady-state levels of hMCPH1 throughout the cell cycle in cultured human cells. HeLa cells were synchronized by nocodazole block and release, and aliquots were taken at two-hour time points in order to assess endogenous levels of hMCPH1, Cyclin B, Cyclin A, p27, and Cdk1 by immunoblotting (supplementary material Fig. S2B). From 2–10 hours after nocodazole release, p27 levels were elevated, and Cyclin A and Cyclin B levels were decreased, consistent with cell-cycle progression into G1. By 10 hours after nocodazole release, p27 levels were decreased, whereas Cyclin A and Cyclin B levels were increased, indicating cell-cycle progression through S, G2, and M-phase. Throughout the time course, hMCPH1 levels remained constant. Taken together, these data suggest that the overall cellular levels of hMCPH1 do not fluctuate in an APC-dependent manner.

### Overexpression of hMCPH1 or dMCPH1-B results in cell-cycle defects

Because APC-mediated degradation of substrates is required for cell-cycle progression, we sought to determine if increasing MCPH1 levels would lead to disruption of cell division. The *Xenopu*s embryo system has been previously used as an in vivo readout of cell cycle progression (Fang et al., 1998a; Ivanovska et al., 2004; McGarry and Kirschner, 1998; Pfleger et al., 2001a; Pfleger et al., 2001b; Rankin et al., 2005). An advantage of the *Xenopus* embryo system is that the non-injected cells act as a negative control within the same embryo.

**Fig. 4.**
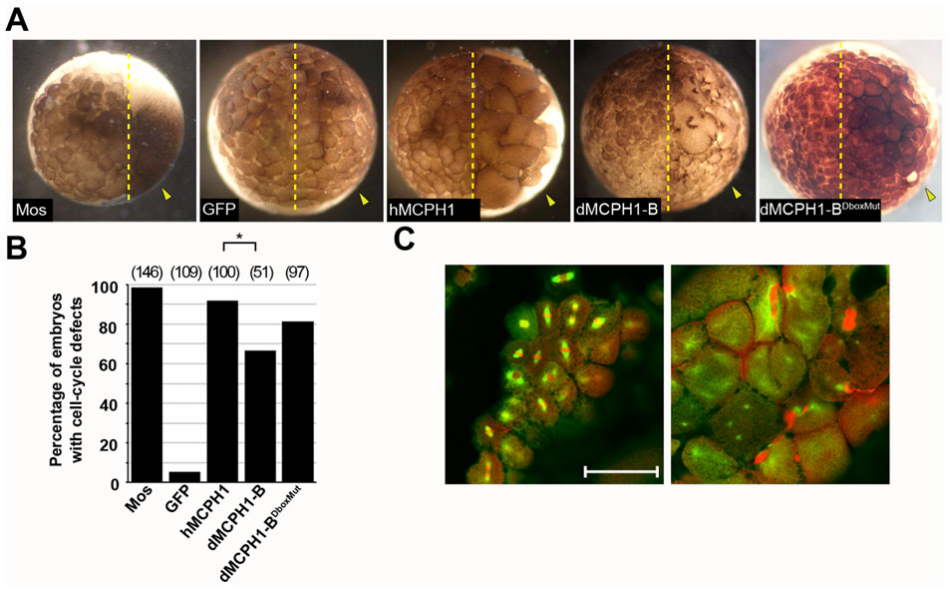
Overexpression of dMCPH1-B or hMCPH1 results in cell-cycle defects. (A) Representative images of whole *Xenopus* embryos fixed four hours after injection of Mos, GFP, full-length human MCPH1 (hMCPH1), dMCPH1-B, or dMCPH1-B^DboxMut^ RNA at the 2–4-cell stage. Arrows indicate injected halves of embryos. (B) Quantification of *Xenopus* embryos displaying cell division defects 4 hours post-injection. Total number of embryos injected is indicated in parentheses. *p<0.005 (C) Confocal sections of the uninjected (left) and injected (right) areas of a representative whole embryo following injection with hMCPH1 mRNA. Microtubules, green; DNA, red. Scale bar: 100 µm.

We tested whether injecting mRNAs encoding hMCPH1 or dMCPH1-B into developing *Xenopus* embryos at the 2–4 cell stage would lead to disruption of cell division. Because Cdh1 is absent in the early embryo, levels of injected MCPH1 should not be regulated by APC, leading to inappropriate activity during these early embryonic cell cycles (Lorca et al., 1998). Injected embryos were allowed to develop, fixed, and assessed for cell division defects (Fig. 4A,B). Mos (a component of cytostatic factor; positive control) is required to maintain metaphase arrest during meiosis II by inhibiting APC activity (Tunquist and Maller, 2003). Injection of Mos mRNA resulted in a block in cell division in the injected half of the embryo. Injection of GFP (negative control) had no observable cell cycle effect on the injected cells. In contrast, 92% of embryos injected with hMCPH1 and 67% of those injected with dMCPH1-B exhibited reduced cell number and increased cell size, likely due to cell-cycle arrest. The levels of the human and *Drosophila* MCPH1 proteins expressed in embryos are nearly equivalent as assessed by immunoblotting (supplementary material Fig. S3). Thus, we attribute the difference in potency between hMCPH1 and dMCPH1-B to be due to differences in sequence identity between the insect and vertebrate proteins. Finally, injections of the D-box mutant of dMCPH1-B also result in embryos with cell cycle arrest (Fig. 4A,B). As expected, levels of the mutant are comparable to that of the wild-type dMCPH1-B protein (not degraded due to the absence of Cdh1 in the early embryo) (supplementary material Fig. S3).

Embryos injected with hMCPH1 were fixed and stained for tubulin and DNA to further examine the cell-cycle defects associated with hMCPH1 overexpression (Fig. 4C). In contrast to the uninjected cells, hMCPH1-injected cells contained abnormal spindle arrangements, free centrosomes, lack of DNA, and/or DNA trapped between daughter blastomeres. These findings are consistent with a previous study in which Sororin, another substrate of APC^Cdh1^, was overexpressed in *Xenopus* embryos (Rankin et al., 2005).

## DISCUSSION

In our DIVEC screen for APC substrates in *Xenopus* egg extract, we identified two candidates: the protein encoded by *CG32982*, a previously uncharacterized *Drosophila* gene, and dMCPH1-B, a splice variant of *Drosophila mcph1*, the homologue of a human microcephaly gene. We show that dMCPH1-B undergoes Cdh1-dependent degradation in *Xenopus* egg extract and not Cdc20-dependent degradation. We show that APC-mediated degradation of dMCPH1 is restricted primarily to the splice variant dMCPH1-B, which contains an N-terminal D-box sequence required for Cdh1-mediated degradation. This restriction may allow for tissue- or developmental-specific regulation of dMCPH1 levels during the cell cycle. Consistent with this idea, we show that dMCPH1 levels are up-regulated in syncytial embryos with reduced APC activity (*mr^1^*/*mr^2^*), a developmental stage in which dMCPH1-B is preferentially expressed. The low level of dMCPH1-C degradation may reflect cryptic APC^Cdh1^ site(s) that is recognized in our optimized system. Alternatively, our system may be missing a co-factor required for efficient turnover of dMCPH1-C by APC^Cdh1^ in *Drosophila* embryos that allows for differential regulation of dMCPH1-B and dMCPH1-C by the APC.

Because dMCPH1-B is preferentially expressed during *Drosophila* syncytial embryogenesis and is down-regulated by the APC, one would predict that dMCPH1-B levels would oscillate throughout the cell cycle during this developmental stage. However, oscillations in total levels of APC substrates, such as mitotic cyclins, are not observed until the later cycles of syncytial embryogenesis (Raff et al., 2002). In fact, localized degradation of Cyclin B by the APC is proposed to control cell-cycle progression during these syncytial cycles (Raff et al., 2002). Thus, it is not surprising that Brunk et al. observed no change in total levels of dMCPH1 during the cell cycles of syncytial embryogenesis (Brunk et al., 2007). It is possible that dMCPH1-B, like Cyclin B, is targeted for degradation in a localized manner.

In vitro ubiquitination assays also revealed that the N-terminal D-box of dMCPH1-B is sufficient for APC^Cdh1^-mediated ubiquitination. The finding that the N-terminal D-box is also not required for APC^Cdh1^-mediated ubiquitination suggests that dMCPH1-B contains additional degrons. This finding is not surprising because many APC substrates have been shown to contain multiple APC-targeting motifs (Min and Lindon, 2012). Although dMCPH1-B contains multiple predicted D-box motifs, we show that deletion of the N-terminal D-box is sufficient to significantly block its Cdh1-dependent degradation in *Xenopus* interphase egg extract. dMCPH1-C also contains many of these putative D-boxes motifs, as well two motifs in the C-terminal region that are not shared with dMCPH1-B. These motifs potentially mediate the low level of degradation in APC^Cdh1^-activated *Xenopus* egg extract.

Two isoforms of human MCPH1 produced by alternative splicing have been previously described and are structurally similar to *Drosophila* dMCPH1-B and C (Gavvovidis et al., 2012). The full-length form of hMCPH1 (used in the current study) contains an N-terminal and two C-terminal BRCT domains, whereas the short form lacks the C-terminal paired BRCT domain region. A previous report has shown that the C-terminal paired BRCT domains of full-length hMCPH1 interact with Cdc27, a subunit of the APC, and the authors hypothesized that hMCPH1 is a substrate of the APC or may regulate APC activity (Singh et al., 2012). In our current study, however, we were not able to observed changes in bulk steady-state hMCPH1 levels in cultured human cells during the cell cycle.

MCPH1 has been shown to be a rapidly evolving gene that exhibits low sequence similarity between homologs (Ponting and Jackson, 2005). Therefore, it is perhaps not surprising that several functions of MCPH1 appear to be species-specific. For example, only hMCPH1 has been shown to regulate condensin II-dependent chromosome condensation (Yamashita et al., 2011). Thus, it is possible that APC-dependent regulation of *Drosophila* MCPH1 is not a conserved feature in humans. Alternatively, similar to the situation with Cyclin B in early embryos of *Drosophila*, levels of hMCPH1 may be regulated locally. Alternatively, the activity of hMCPH1 could be regulated via its binding partners/effectors. Indeed, binding partners, SET/Phosphatase Inhibitor 2 and E2F1, are potential or known APC substrates, respectively (Brautigan et al., 1990; Budhavarapu et al., 2012; Leung et al., 2011; Peart et al., 2010; Yang et al., 2008). Thus, the regulation of these two MCPH1 binding partners by the APC could serve as a mechanism to regulate MCPH1 activity in a cell cycle-dependent manner in vertebrates.

We show herein that overexpression of either hMCPH1 or dMCPH1-B in *Xenopus* embryos, an assay that has been previously used to characterize important cell-cycle regulators, leads to cell-cycle defects (Fang et al., 1998a; Ivanovska et al., 2004; McGarry and Kirschner, 1998; Pfleger et al., 2001a; Pfleger et al., 2001b; Rankin et al., 2005). This finding suggests that tight regulation of the levels of MCPH1 may be required for proper cell-cycle progression. Because hMCPH1 is known to negatively regulate mitotic entry and chromosome condensation, the cell-cycle defects we observe in *Xenopus* embryos overexpressing MCPH1 may be due to misregulation of these processes (Alderton et al., 2006; Tibelius et al., 2009; Trimborn et al., 2006; Yamashita et al., 2011). Although MCPH1 has been implicated in many cellular processes, regulation of its activity is not well understood. Future studies to elucidate how the activities and/or levels of MCPH1 are controlled will be important to fully understand how this evolutionarily conserved, highly evolving protein functions in regulating critical processes within the developing organism.

## Supplementary Material

Supplementary Material
