# Supplementary material for: The Drosophila MCPH1-B isoform is a substrate of the APCCdh1 E3 ubiquitin ligase complex
Source: Biol Open. 2014 Jun 27;3(7):669–76. doi: 10.1242/bio.20148318 (PMC4154303; doi:10.1242/bio.20148318)
Supplement: Supplementary Material [file supp_3_7_669__index.html]

The Drosophila MCPH1-B isoform is a substrate of the APCCdh1 E3 ubiquitin ligase complex — The Drosophila MCPH1-B isoform is a substrate of the APCCdh1 E3 ubiquitin ligase complex — Supplementary Material 

# The *Drosophila* MCPH1-B isoform is a substrate of the APCCdh1 E3 ubiquitin ligase complex

## bio.20148318 Supplementary Material

**Files in this Data Supplement:**

- Supplementary Material - Sarah G. Hainline et al. doi: 10.1242/bio.20148318
