# Supplementary material for: The Drosophila MCPH1-B isoform is a substrate of the APCCdh1 E3 ubiquitin ligase complex
Source: Biol Open. 2014 Jun 27;3(7):669–76. doi: 10.1242/bio.20148318 (PMC4154303; doi:10.1242/bio.20148318)
Supplement: Supplementary Material [file supp_bio.20148318_bio.20148318-s1.pdf]

Biology Open (2014) 000, 1–8 doi:10.1242/bio.20148318

Supplementary Material

Sarah G. Hainline et al. doi: 10.1242/bio.20148318

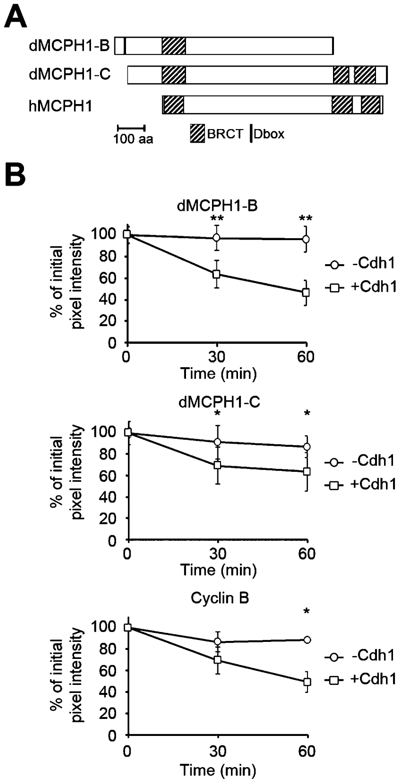

Fig. S1. APC regulates stability of dMCPH1-B and dMCPH1-C. (A) Schematic representation of dMCPH1-B, dMCPH1-C, and hMCPH1. (B) Quantitation of pixel intensity of autoradiogram in Fig. 2B. Percent of initial pixel intensity was plotted over time for radiolabeled dMCPH1-B, dMCPH1-C, or Cyclin B incubated in *Xenopus* interphase egg extract in the absence or presence of Cdh1. **$p<0.005$, *$p<0.05$.

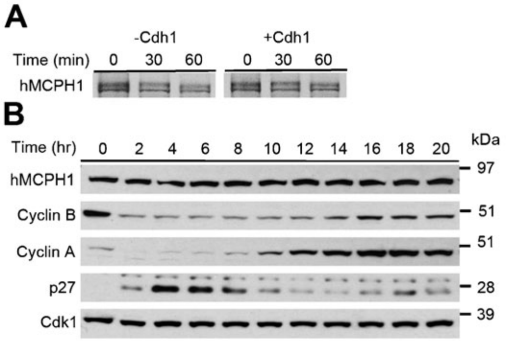

Fig. S2. APC does not regulate the stability of hMCPH1. (A) Autoradiogram of radiolabeled hMCPH1 incubated in *Xenopus* interphase egg extract in the absence or presence of Cdh1. (B) Levels of hMCPH1 do not notably fluctuate in a cell cycle-dependent manner. Immunoblot analysis of hMCPH1, Cyclin B, Cyclin A, p27, and Cdk1 in lysates derived from synchronized HeLa cells 0–20 hours after nocodazole release. Degradation of Cyclin A and B occurs in prophase and metaphase, respectively, whereas degradation of p27 marks late G1/S.

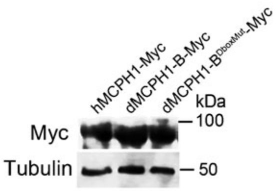

Fig. S3. *Xenopus* embryos express hMCPH1-Myc, dMCPH1-B-Myc, and dMCPH1-B^{DboxMut}-Myc at similar levels. Immunoblot for Myc and tubulin (loading control) of lysates derived from *Xenopus* embryos after injection with mRNA encoding hMCPH1-Myc, dMCPH1-B-Myc, or dMCPH1-B^{DboxMut}-Myc.
